# Association between behavioural risk factors for hypertension and concordance with the Dietary Approaches to Stop Hypertension dietary pattern among South Asians in the Mediators of Atherosclerosis in South Asians Living in America (MASALA) study

**DOI:** 10.1017/jns.2025.8

**Published:** 2025-03-05

**Authors:** Bridget Murphy Hussain, Andrea L. Deierlein, Alka M. Kanaya, Sameera A. Talegawkar, Joyce A. O’Connor, Meghana D. Gadgil, Belinda L. Needham, Yong Lin, Niyati Parekh

**Affiliations:** 1 Public Health Program, Egan School of Nursing and Health Studies, Fairfield University, Fairfield, CT, USA; 2 Public Health Nutrition, School of Global Public Health, New York University, New York, NY, USA; 3 Division of General Internal Medicine, Department of Medicine, University of California, San Francisco, San Francisco, CA, USA; 4 Departments of Exercise and Nutrition Sciences and Epidemiology, Milken Institute School of Public Health at The George Washington University, Washington, DC, USA; 5 Department of Epidemiology and Center for Social Epidemiology and Population Health, University of Michigan, Ann Arbor, MI, USA; 6 Department of Biostatistics and Epidemiology, School of Public Health, Rutgers University, Newark, NJ, USA; 7 Department of Population Health, New York University Langone Health, New York, NY, USA; 8 Rory Meyers School of Nursing, New York University Langone Health, New York, NY, USA

**Keywords:** South Asian, Dietary Approaches to Stop Hypertension, Dietary intake, Cardiovascular disease, ACC/AHA, American College of Cardiology and American Heart Association, ASCVD, atherosclerotic cardiovascular disease, BMI, body mass index, DASH, dietary approaches to stop hypertension, MASALA, mediators of atherosclerosis, U.S., United States

## Abstract

South Asians are among the fastest-growing immigrant population group in the United States (U.S.) with a unique disease risk profile. Due in part to immigration and acculturation factors, South Asians engage differently with behavioural risk factors (e.g. smoking, alcohol intake, physical activity, sedentary behaviour, and diet) for hypertension, which may be modified for the primary prevention of cardiovascular disease. Using data from the Mediators of Atherosclerosis in South Asians Living in America cohort, we conducted a cross-sectional analysis to evaluate the association between behavioural risk factors for cardiovascular disease and diet. We created a behavioural risk factor score based on smoking status, alcohol consumption, physical activity, and TV watching. We also calculated a Dietary Approaches to Stop Hypertension (DASH) dietary score based on inclusion of relevant dietary components. We used both scores to examine the association between engaging with risk factors for hypertension and the DASH diet among a cohort of South Asian adults. We found that participants with 3–4 behavioural risk factors had a DASH diet score that was 3 units lower than those with no behavioural risk factors (aβ: –3.25; 95% CI: –4.28, –2.21) and were 86% less likely to have a DASH diet score in the highest category compared to the lowest DASH diet score category (aOR: 0.14; 95% CI: 0.05, 0.37) in the fully adjusted models. These findings highlight the relationship between behavioural risk factors for hypertension among South Asians in the U.S.

## Introduction

Hypertension is a leading risk factor for the early development of atherosclerotic cardiovascular disease (ASCVD).^(1,2)^ Modifiable behavioural risk factors for hypertension include smoking, alcohol intake, physical activity, and sedentary behaviour.^(3)^ The American College of Cardiology and American Heart Association (ACC/AHA) specify that never smoking, minimising alcohol intake, engaging in at least 150 minutes of physical activity per week, and consuming a healthy diet are essential lifestyle characteristics for the primary prevention of cardiovascular disease.^(3)^ Reducing sedentary behaviour (defined as sitting, reclining, and watching television (TV)) is additionally included as a subgoal in the guidelines’ recommendations for physical activity.^(3)^

The Dietary Approaches to Stop Hypertension (DASH) diet^(4)^ is a pattern of eating characterised by increased consumption of fruits, vegetables, legumes and nuts, that has been highlighted by the ACC/AHA due to its proven ability to reduce the risk of hypertension.^(3)^ Previous research has emphasised the impact of DASH on incident hypertension,^(5–7)^ but studies examining the relationship between behavioural risk factors for hypertension and adherence to the DASH dietary pattern are needed to define the interdependence between modifiable lifestyle behaviours that can reduce risk for hypertension and prevent development of ASCVD.

According to the ACC/AHA guidelines, South Asian ancestry is considered a risk-enhancing clinical factor in the ASCVD risk estimate calculation.^(3)^ This is due to both biological (e.g. high central adiposity and greater prevalence of type 2 diabetes) and nonbiological mechanisms.^(8)^ Nonbiological mechanisms include health behaviours that result in relationship to acculturation,^(8)^ defined as the process of cultural and psychological change when members from multiple cultural groups interact.^(9,10)^ As South Asians are among the fastest-growing immigrant population group in the United States (U.S.),^(8)^ it is essential to understand the behavioural risk factors that may place South Asian immigrants at increased risk for ASCVD. A recent study among South Asian immigrants in Canada found that recent immigrants were more likely to be physically inactive compared to established immigrants, while also more likely to consume high-calorie packaged foods due to increased access to supermarkets.^(11)^ More studies are needed that characterise features of diet and lifestyle factors of South Asian immigrants in the U.S., with particular attention to a DASH-style dietary pattern that is inclusive of both traditional and western food items and ingredients.^(12)^

To our knowledge, previous studies have not examined DASH diet intake among South Asian immigrant populations. Using data from the Mediators of Atherosclerosis in South Asians Living in America (MASALA) study,^(13)^ we examined the association between behavioural risk factors for hypertension (i.e. ever smoking, consuming alcohol, low physical activity, and high TV watching) and DASH diet adherence. We hypothesised that participants with more behavioural risk factors would have a lower DASH diet adherence.

## Methods

### Study population

The MASALA study is an ongoing community-based cohort study of South Asian Americans aged 40–84 years, free from cardiovascular disease at enrolment. A detailed description of the study rationale, design, and methods has been described elsewhere.^(13)^ Briefly, adults who self-identified as South Asian with three or more grandparents born in Bangladesh, India, Nepal, Pakistan, or Sri Lanka were recruited from the greater Chicago area near Northwestern University (NWU) and the San Francisco Bay area at University of California, San Francisco (UCSF). The primary objective of the MASALA study is to prospectively study factors related to ASCVD among recruited participants who were free of cardiovascular disease at exam 1. The protocol was approved by both the NWU and UCSF Institutional Review Boards (IRB),^(13)^ and the current analyses were approved by the New York University IRB (IRB-FY2021-5009).

### Analytical dataset

**Figure 1.**
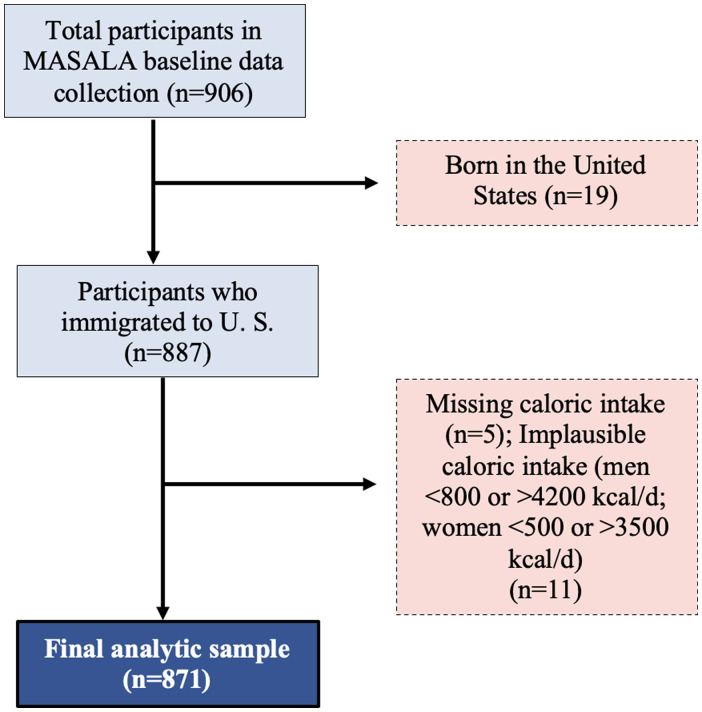
Creation of the final analytical data set from the Mediators of Atherosclerosis in South Asians Living in America cohort (2010–2013). Examination 1 was used as baseline in the present analyses.

Participants with baseline data collected at exam 1 (baseline, 2010–2013) were eligible for inclusion in the analytical sample (*n* = 906). As 98% of participants were born outside the U.S., we excluded those who were born in the U.S. (*n* = 19). We also excluded participants with missing dietary data or implausible caloric intake (<800 kilocalories/day or >4,200 kilocalories/day for men and <500 kilocalories/day or >3500 kilocalories/day for women; *n* = 16).^(14)^ The final analytical sample included 871 participants (Figure 1).

### Data collection

#### Behavioural risk factors for hypertension

We examined non-dietary behavioural risk factors for hypertension to examine their association with diet. Smoking status was reported as never, former, or current smoker. Alcohol consumption was collected based on average consumption per week (<1 drink per week, 1–7 drinks per week, >7 drinks per week). Physical activity was assessed using the Typical Week’s Physical Activity Questionnaire^(15)^ and categorised using criteria defined by Life’s Simple Seven (poor (no activity), intermediate (1–149 minutes of moderate or 1–74 minutes of vigorous activity per week), ideal (≥150 minutes of moderate or ≥75 minutes of vigorous activity per week)).^(16,17)^ Sedentary behaviours were assessed using minutes of television (TV) watching per week assessed using the Typical Week’s Physical Activity Questionnaire.^(15)^

#### Dietary data

The Study of Health Assessment and Risk in Ethnic groups (SHARE) food frequency questionnaire (FFQ)^(18)^ was administered by MASALA study staff to participants. The SHARE FFQ is a 163-item tool validated to assess dietary intake during the previous year of South Asian adults living in Canada and is validated for use among South Asians living in North America. Food and beverage intakes were assessed by frequency of consumption (i.e. per day, per week, per month, or per year) and usual serving sizes (i.e. cups, tablespoons, ounces, or millilitres) to estimate average daily quantities with total energy intake (kilocalories/day).^(18)^

#### Sociodemographic data

Participants self-reported biological sex (male/female); annual family income (response choices <$40,000 per year, $40,000–$75,000 per year, $75,000–$100,000 per year, and >$100,000 per year); income was dichotomised as >$100,000 per year and ≤$100,000 per year; educational attainment dichotomised as achieved bachelor’s degree (yes or no). Per cent of life lived in the U.S. was determined by dividing number of years living in the U.S. into participants age (continuous). Acculturation was determined using twelve indicators of acculturation inclusive of following South Asian traditions, such as performing religious ceremonies or rituals, fasting, and following a traditional South Asian diet, and lifestyle behaviours, such as family shopping. Latent class analysis was used to identify three acculturation strategies including: (1) ‘separation’, characterised by a preference for South Asian culture over U.S. culture; (2) ‘assimilation’, characterised by a preference for U.S. culture over South Asian culture; and (3) ‘integration’, characterised by a similar preference for South Asian and U.S. culture.^(19)^ A detailed description of the methodology used to identify acculturation strategies can be found in previously published work.^(9)^

### Data analysis

#### Creation of the behavioural risk factors score

**Figure 2.**
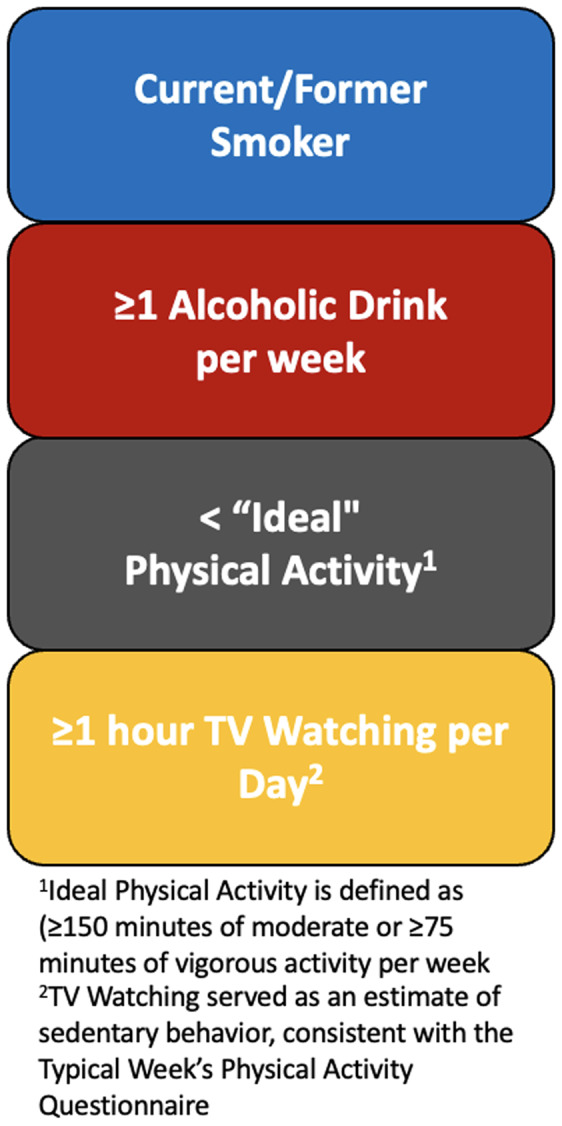
Criteria for creating the behavioural risk factor score.

**Figure 3.**
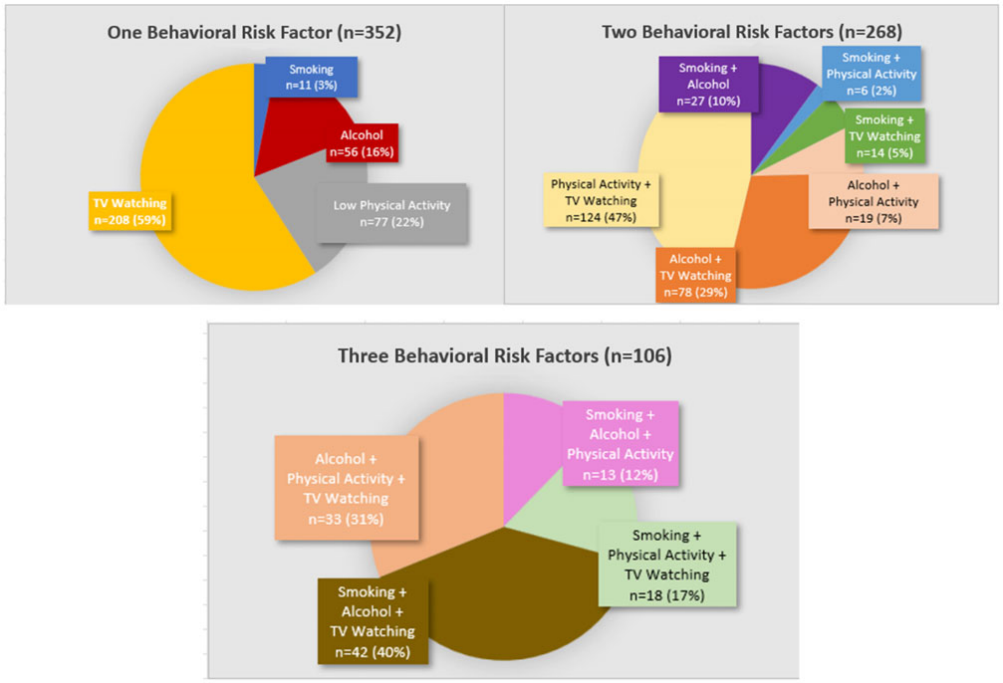
Participants with 1–3 behavioural risk factors (*n* = 726 (83% of analytic sample)). Top Left: Participants with one behavioural risk factor (i.e. they did not have the other three behavioural risk factors); Top Right: Participants with two behavioural risk factors; Bottom: Participants with three behavioural risk factors; not pictured: Participants with no behavioural risk factors, or all four behavioural risk factors.

We created a behavioural risk factor score by tabulating the number of risk factors, including smoking, alcohol, physical activity, and TV watching (Figure 2). Each variable was dichotomised for higher risk versus lower risk (i.e. current or former smoker score = 1, never smoker score = 0; ≥1 alcoholic drink per week score = 1, <1 alcoholic drink per week score = 0; poor or intermediate physical activity score = 1, ideal physical activity score = 0). Consistent with previous research using the Typical Week’s Physical Activity Questionnaire to assess sedentary behaviour,^(20)^ TV watching was dichotomised by <1 hour/day (score = 0) and ≥1 hour/day (score = 1). Participants had a theoretical score range from 0 to 4. As only 15 participants (1.7%) had a score of 4, behavioural risk factor score was assessed by no behavioural risk factors, one behavioural risk factor, two behavioural risk factors, and 3–4 behavioural risk factors. Figure 3 shows the distribution of behavioural risk factors.

#### Creation of the DASH diet score

Using the SHARE FFQ data, we computed the total and component DASH dietary concordance score using the Fung *et al.* method to quantify adherence to the dietary pattern.^(21)^ This method is also used by the AHA for calculating the Life’s Essential 8 diet score.^(22)^ The total DASH score is based on scores for eight components: high intake of (1) fruit, (2) vegetables, (3) nuts/legumes, (4) low-fat dairy products, and (5) whole grains, and low intake of (6) sodium, (7) sugar-sweetened beverages, and (8) red or processed meats. Participants were classified into quintiles according to intake of each component. Sodium, sugar-sweetened beverage intake, and red or processed meat were reverse scored, such that higher intakes correspond to lower scores (Supplementary Table 1). The theoretical total DASH score may range from 8 (low concordance) to 40 (high concordance). Consistent with previous studies, total DASH score was categorised as low (≤20), moderate (21–28), and high (≥29) concordance for analyses.^(21,23)^

### Statistical analyses

Descriptive statistics (mean and standard deviation for continuous variables; count and per cent for discrete variables) for demographic characteristics were calculated for the total population, by DASH dietary score category, and by behavioural risk factor score. For continuous variables, test of linear trends across groups was assessed using analysis of variance. For categorical variables, between-group differences were evaluated using Pearson’s Chi-square.

Covariates were assessed for retention in the multivariable models using directed cyclic graphs^(24,25)^ to help identify the presence of potential confounding in the relationship between the behavioural risk factors and DASH diet score. Educational attainment and income were highly correlated (correlation ρ = –0.30; P < 0.0001). There were 26 (3%) participants missing household income; therefore, we adjusted for educational attainment. Models include age adjustment and multivariable adjustment. Final models were adjusted for age, sex, per cent of life lived in the U.S., education, and acculturation strategies (Model 1), with the additional adjustment for daily energy intake (Model 2).

Behavioural risk factors score was assessed as a categorical variable. DASH diet was assessed by continuous score and by concordance category divided as low (20)≤, medium (21–28), and high (29)≥. We evaluated associations of behavioural risk factors score and DASH dietary concordance continuously using linear regression and categorically using multinomial logistic regression. Analyses were performed using the statistical software package Stata version 16.1^(26)^ and all statistical tests were two-sided with significance level set at P < 0.05.

## Results

### Sample characteristics

Baseline characteristics of the sample, total and stratified by DASH diet score category, are shown in Table 1. The mean age of participants was 55.5 years (SD = 9.3) and 47% were women (*n* = 408). On average, participants lived about half of their life in the U.S. (mean = 48.3% life lived in the U.S.; SD = 16.5 years), and a majority of participants had a bachelor’s degree or higher (*n* = 764; 88%) and family income >$100,000 per year (*n* = 534; 63%). Acculturation strategies were associated with DASH diet score, with a greater proportion of participants in the ‘integration’ group in the highest DASH diet category (*n* = 97 (58.1%); P < 0.0001). Higher DASH diet score was associated with higher energy intake, lower prevalence of ever smoking, lower alcohol intake, and higher physical activity in the bivariate analysis, but not with minutes of TV watching per week.

**Table 1.** Participant characteristics at exam 1 by group of Dietary Approaches to Stop Hypertension diet score, among South Asian adults in the Mediators of Atherosclerosis in South Asians Living in America study, 2010–2013 (*n* = 871)

Characteristic	Total study population (*n* = 871)	DASH score 13–20 (lowest) (*n* = 162)	DASH score 21–28 (*n* = 542)	DASH score 29–35 (highest) (*n* = 167)	P *value*
**Sociodemographic information**					
Age (y) (mean (SD))	55.5 (9.3)	53.6 (9.6)	56.1 (9.4)	55.6 (8.9)	0.02
Women (*n* (%))	408 (46.8)	51 (31.5)	256 (47.2)	101 (60.5)	<0.0001
Percent of life lived in US (mean (SD))	48.3 (16.5)	50.9 (16.7)	48.2 (16.7)	46.1 (15.3)	0.03
Bachelor’s degree or higher (*n* (%))	764 (87.7)	132 (81.5)	479 (88.4)	153 (91.6)	0.02
Income >$100K (*n* (%))	534 (63.2)	100 (62.1)	320 (61.7)	114 (69.1)	0.22
Acculturation strategies (*n* (%)					<0.0001
Assimilation	263 (30.2)	75 (46.3)	148 (27.3)	40^(24)^	
Separation	157^(18)^	25 (15.4)	102 (18.8)	30^(18)^	
Integration	451 (51.8)	62 (38.3)	292 (53.9)	97 (58.1)	
**Diet and behavioural factors**					
Energy intake (mean (SD))	1,687.8 (510.4)	1,667.8 (537.8)	1,653.9 (508.1)	1,817.4 (471.3)	0.001
Smoked ever (former/current) (*n* (%))	146 (16.8)	44 (27.2)	87 (16.1)	15 (9.0)	<0.0001
≥1 drink alcohol per week (*n* (%))	283 (32.5)	81 (50.0)	163 (30.1)	29 (23.4)	<0.0001
Physical activity^a^ (*n* (%))					<0.0001
Poor	134 (15.4)	38 (23.5)	82 (15.1)	14 (8.4)	
Intermediate	171 (19.6)	43 (26.5)	102 (18.8)	26 (15.6)	
Ideal	566 (65.0)	81 (50.0)	358 (66.1)	127 (76.1)	
TV minutes/week (Median (IQR))	420 (630)	420 (630)	520 (630)	420 (660)	0.63
≥1 hour of TV per day	532 (51.1)	101 (62.4)	328 (60.5)	103 (61.7)	0.90

a Typical Week’s Activity Survey (Poor indicates no activity; Intermediate indicates 1–149 minutes of moderate or 1–74 minutes of vigorous activity per week; Ideal indicates ≥150 minutes of moderate or ≥75 minutes of vigorous activity per week.

P-values were estimated by analysis of variance for continuous variables and Pearson’s chi-square for categorical variables.

### Sociodemographic associations

Table 2 presents the bivariate associations between sociodemographic characteristics and number of behavioural risk factors for hypertension. Participants with 3–4 behavioural risk factors were older and had lived in the U.S. for a greater percent of their lives compared to participants with no behavioural risk factors. A greater proportion of women had 3–4 behavioural risk factors than men (*n* = 105; 87%). A greater proportion of participants with higher socioeconomic status (i.e. those who held their bachelor’s degree or greater had an income >$100,000 per year) had no behavioural risk factors (*n* = 125 (95%); *n* = 92 (72%); P = 0.03 for both). A higher proportion of participants in the integration group had no behavioural risk factors (*n* = 76 (58.5%); P = 0.02), compared to those in the assimilation and separation groups. There was a bivariate association between number of behavioural risk factors and total energy intake, continuous DASH diet score, and DASH diet score category. Table 3 shows the correlation between each of the behavioural risk factors and DASH diet score category. Smoking was highly correlated with alcohol intake (*rho* = 0.5524; P < 0.0001) and with DASH diet score category (*rho* = –0.1492; P < 0.0001). Alcohol intake was correlated with physical activity (*rho* = –0.1664; P = 0.003) and DASH diet score category (*rho* = –0.1740; P < 0.0001). DASH diet score category was also associated with physical activity (*rho* = –0.1674; P < 0.0001).

**Table 2.** Participant characteristics by behavioural risk factors, among South Asian adults in the Mediators of Atherosclerosis in South Asians Living in America study, 2010–2013 (*n* = 871)

Characteristic	Total study population (*n* = 871)	No behavioural risk factors(*n* = 130)	One behavioural risk factor (*n* = 352)	Two behavioural risk factors (*n* = 268)	3–4 behavioural risk factors (*n* = 121)	P *value*
**Demographic information**						
Age (y) (mean (SD))	55.5 (9.3)	52.7 (8.9)	56.2 (9.3)	54.7 (9.4)	58.6 (8.8)	<0.0001
Women (*n* (%))	408 (46.8)	59 (45.4)	162 (46.0)	137 (51.1)	105 (86.8)	<0.0001
Percent of life lived in US (mean (SD))	48.3 (16.5)	44.2 (15.0)	49.3 (16.9)	49.0 (17.1)	48.0 (15.2)	0.02
Bachelor’s degree or higher (*n* (%))	764 (87.7)	124 (95.4)	306 (86.9)	229 (85.5)	105 (86.8)	0.03
Income >$100K (*n* (%))	534 (63.2)	92 (72.4)	198 (58.6)	172 (65.7)	72 (61.0)	0.03
Acculturation strategies (*n* (%)						0.02
Assimilation	263 (30.2)	32 (24.6)	90 (25.6)	96 (35.8)	45 (37.2)	
Separation	157 (18)	22 (16.9)	71 (20.2)	49 (18.3)	15 (12.4)	
Integration	451 (51.8)	76 (58.5)	191 (54.3)	123 (45.9)	61 (50.4)	
**Diet and behavioural factors**						
Energy intake (mean (SD))	1,687.8 (510.4)	1,619.1 (534.1)	1,668.3 (447.1)	1,698.3 (525.7)	1,795.5 (531.7)	0.04
DASH diet score (mean (SD))	24.6 (4.4)	26.0 (4.1)	25.4 (4.1)	23.8 (4.1)	22.4 (4.6)	<0.0001
DASH diet score (*n* (%))						<0.0001
Category 1: 13–20	162 (18.6)	13 (10.0)	42 (11.9)	62 (23.1)	45 (37.2)	
Category 2: 21–28	542 (62.2)	84 (64.6)	225 (63.9)	170 (63.4)	63 (52.1)	
Category 3: 29–35	167 (19.2)	33 (25.4)	85 (24.2)	36 (13.4)	13 (10.7)	

P-values were estimated by analysis of variance for continuous variables and Pearson’s chi-square for categorical variables.

**Table 3.** Correlations^a^ between behavioural risk factors, among South Asian adults in the Mediators of Atherosclerosis in South Asians Living in America study (*n* = 871)

	Smoking (current/former)	≥1 alcoholic drink/week	<Ideal Physical Activity	≥1 hour of TV/day
≥1 alcoholic drink/week	0.5524**	–	–	–
<Ideal Physical Activity	0.0108	–0.1664*	–	–
≥1 hour of TV/day	–0.0021	–0.0403	0.0300	–
DASH diet score category	–0.1492**	–0.1740**	–0.1674**	–0.0040

a Spearman’s *rho* for correlations with DASH diet score category; Tetrachoric *rho* for all other correlations.

*P < 0.01; **P < 0.0001.

### Behavioural risk factor score and DASH diet score

Age- and multivariable-adjusted models assessing the relationship between behavioural risk factors and DASH diet score are presented in Table 4. Participants with 3–4 behavioural risk factors had a DASH diet score that was approximately 3 units lower than those with no behavioural risk factors (aβ: –3.25; 95% Confidence Interval (CI): –4.28, –2.21; P < 0.0001) in the model adjusted for age, sex, per cent of life lived in the U.S., education, acculturation strategies, and energy intake per day. Similarly, participants with 3–4 behavioural risk factors were 77% less likely and 86% less likely to be in the medium or high DASH diet score category, respectively, when compared to participants with no behavioural risk factors (low vs. medium DASH diet score category: aOR: 0.23; 95% CI: 0.11, 0.49; P < 0.0001; low vs. high DASH diet score category: aOR: 0.14; 95% CI: 0.05, 0.37; P < 0.0001).

**Table 4.** Age-adjusted and multivariable-adjusted Dietary Approaches to Stop Hypertension (DASH) diet score by number of behavioural risk factors, among South Asian adults in the Mediators of Atherosclerosis in South Asians Living in America study (*n* = 871)

	No behavioural risk factors (*n* = 130)	One behavioural risk factor (*n* = 352)		2 behavioural risk factors (*n* = 268)		3–4 behavioural risk factors (*n* = 121)		
	Reference	β	95% CI	β	95% CI	β	95% CI	P *_trend_^a^*
DASH diet score (continuous)								
Age adjusted	0.00	–0.77	–1.62, 0.07	–2.31	–3.19, –1.43	–3.98	–5.02, –2.93	<0.0001
Model 1^b^	0.00	–0.55	–1.37, 0.28	–1.85	–2.71, –0.99	–3.07	–4.11, –2.02	<0.0001
Model 2^c^	0.00	–0.64	–1.46, 0.17	–1.99	–2.83, –1.14	–3.25	–4.28, –2.21	<0.0001
	Reference	RR	95% CI	RR	95% CI	RR	95% CI	P *_trend_^a^*
DASH diet score (low (13–20) vs. medium (21–28))								
Age adjusted	1.00	0.73	0.37, 1.43	0.39	0.20, 0.75	0.17	0.08, 0.34	<0.0001
Model 1^b^	1.00	0.84	0.42, 1.68	0.49	0.25, 0.96	0.24	0.11, 0.50	<0.0001
Model 2^c^	1.00	0.83	0.42, 1.66	0.49	0.25, 0.96	0.23	0.11. 0.49	<0.0001
DASH diet score (low (13–20) vs. high (29–35))								
Age adjusted	1.00	0.71	0.33, 1.49	0.21	0.10, 0.45	0.09	0.04, 0.22	<0.0001
Model 1^b^	1.00	0.86	0.40, 1.86	0.29	0.13, 0.63	0.16	0.06, 0.41	<0.0001
Model 2^c^	1.00	0.81	0.37, 1.76	0.26	0.12, 0.58	0.14	0.05, 0.37	<0.0001

SE, standard error; CI, confidence interval.

a P-trend calculated by unadjusted linear regression, using total behavioural risk factor score (0–4) as a continuous covariate.

b Model 1: Adjusted for age, gender (men/women), per cent life lived in the U.S., education (≥Bachelors/<Bachelors), acculturation (assimilation, separation, integration).

c Model 2: Model 1 + energy (kcal/d).

### Individual risk factors and DASH diet score

Supplementary Tables 2–5 present models assessing the relationship between each of the individual behavioural risk factors for hypertension (i.e. smoking, alcohol intake, physical activity level, and TV watching) and the DASH diet score. Current and former smokers had a DASH diet score that was approximately 1 unit lower than participants who reported never smoking (aβ: –1.25; 95% CI: –2.03, –0.48; P = 0.002) in the fully adjusted model. The association between ever smoking and DASH diet score category was not significant in the fully adjusted models (Supplementary Table 2). Participants who reported consuming one alcoholic drink or more per week had a DASH diet score that was 1 unit lower than those consuming less than one alcoholic drink per week (aβ: –1.18; 95% CI: –1.82, –0.55; P < 0.0001) and were 58% less likely to have a DASH diet score in the highest category (aOR: 0.42; 95% CI: 0.24, 0.75; P = 0.003) in the fully adjusted models (Supplementary Table 3). Participants who met criteria for ideal weekly physical activity had a DASH diet score that was 2 units higher than those who reported no weekly physical activity (aβ: 2.23; 95% CI: 1.46, 2.99; P < 0.0001) and were 6.15 times more likely to have a DASH diet score in the highest category (aOR: 6.15; 95% CI: 2.94, 12.88; P < 0.0001) in the fully adjusted models (Supplementary Table 4). There was not a significant association between TV watching and DASH diet score (Supplementary Table 5).

## Discussion

In this study, we investigated the association between behavioural risk factors for hypertension and the DASH diet among South Asian immigrants in the U.S. We found that participants with more behavioural risk factors had lower overall DASH diet score and were less likely to be in the highest DASH diet score category. Our study provides greater evidence that there is an association between behavioural factors and diet, which better characterises the relationship between risk factors for hypertension and ASCVD. While each behavioural risk factor will contribute differently to hypertension and ASCVD risk, our findings will help to direct opportunities for prevention efforts by clinicians and policymakers for this high-risk immigrant population. To our knowledge, this is the first study assessing the cluster of established behavioural risk factors for hypertension and their association with the DASH diet among South Asian immigrants in the U.S.

Previous research has predominantly assessed the relationship between individual behavioural risk factors and diet. Consistent with our results, one cross-sectional study examined the relationship between smoking status and several different indices of diet and found that DASH diet score was lower among heavy smokers compared to participants who reported never smoking.^(27)^ Similarly, a cross-sectional study of adults in China found that participants with greater knowledge of the deleterious effects of smoking and less smoking practices had better diet quality when compared with those with less smoking-related knowledge.^(28)^ Increased alcohol intake has consistently been linked to higher overall energy intake,^(29)^ and a recent systematic review of studies examining the association between alcohol consumption and diet among adults found that alcohol intake was linked to difference in macronutrient intakes, with alcohol linked to greater intake of fat and protein, as well as greater intake of refined carbohydrates.^(30)^ Several intervention studies of non-Hispanic White and African American adults have examined the effect of combining the DASH diet with physical activity to reduce risk of cardiovascular-related outcomes,^(31–33)^ finding the combined effect of physical activity and diet yield more positive outcomes in the prevention or delay of disease onset.

Few studies examining the relationship between diet and disease risk have been conducted among South Asian adults. A 2002 diet intervention trial among adult men and women in India found that participants who consumed more fruit, vegetables, legumes, walnuts and almonds had a reduction in cardiac deaths and non-fatal myocardial infarctions compared to controls, as well as a reduction in cardiac risk factors, such as serum cholesterol.^(34)^ Similarly, a systematic review of dietary patterns in India identified an association between higher fruit, vegetable, and pulse (i.e. legumes) intake and lower risk for hypertension.^(35)^ Cross-sectional studies examining the relationship between dietary intake and hypertension have been conducted among adults living in India and Pakistan. One study in India found that participants with greater dietary diversity (scoring comprised of grain, vegetable, fruit, legume, non-vegetarian, milk and milk products, sugar, and solid fat/oil intake) had a lower prevalence of hypertension.^(36)^ A similar study conducted among Pakistani urban adults found that participants with higher seafood and yoghourt intake had lower prevalence of hypertension, though they did not find an association between hypertension and dietary patterns characterised by fat and sweet or fruit and vegetable intake.^(37)^ These previous studies among South Asian adult populations are consistent with the DASH dietary components and their impact on cardiovascular-related health, though none have directly assessed the relationship between behavioural risk factors for hypertension and diet.

Interestingly, both the DASH diet score and the behavioural risk factor score were associated with acculturation strategies among this sample of South Asian Americans. These findings are consistent with other work using data from MASALA cohort, which found that participants with stronger traditional cultural beliefs and practices were more likely to consume the dietary pattern characterised by fried snacks, sweets, and high-fat dairy, whereas those with weaker traditional beliefs had a more varied diet with lower fried snacks, sweets, and high-fat dairy intake.^(38)^ These findings indicate that South Asian immigrants who follow an ‘integration’ strategy that combines traditional South Asian beliefs and practices with new, American ones, may support better health behaviours overall when compared to separation and assimilation strategies.

The creation of a behavioural risk factor score illustrates a dose-like response in the association between behavioural risk factors for hypertension and the DASH diet. A previous study of Dutch adults similarly examined the combination of behaviours including smoking, nutrition, alcohol consumption and physical activity, finding that participants who had a combination of unhealthy diet, excessive drinking, and physical inactivity had lower self-rated health.^(39)^ Another study using data from the National Health and Nutrition Examination Survey (2005, 2006) clustered lifestyle behaviours and found that diet quality and physical activity was associated with all-cause mortality.^(40)^ Similarly, a prospective cohort study of Australian adults found that the combination of physical inactivity, prolonged sitting, smoking, and high alcohol intake were all associated with increased all-cause mortality.^(41)^ Taken together, the results of this previous research indicate that combinations of lifestyle risk behaviours in aggregate may increase morbidity and mortality more than one isolated behaviour. Moreover, examining the interdependent nature of these behavioural risk factors and their impact on diet is essential to fully characterising the diet-disease relationship.

High physical activity levels and high sedentary behaviour (defined as high TV watching) were not mutually exclusive. More than half of the study sample (*n* = 342; 62.3%) met criteria for ideal levels of physical activity yet watched one hour or more of TV per day, indicating high sedentary behaviour alongside desirable levels of physical activity. There were not significant differences in median number of minutes of TV watching per week by physical activity level (P = 0.153). This is supported in a previous report that call for the inclusion of limiting sedentary behaviour in clinical guidelines for disease prevention,^(42)^ as sedentary behaviour is a modifier of the positive effects of physical activity on all-cause mortality.^(43)^ At present, the AHA does not include limiting sedentary behaviour as one of their essential recommendations for heart health.^(22)^

The findings from this study should be interpreted considering some limitations. The behavioural risk factors were dichotomised for scoring. It should be noted that there is a dose response with smoking, alcohol intake, physical activity, and sedentary behaviour that is not fully captured using specific cut-off points. While we did use evidence-based, discrete cut-off guidelines, these behavioural risk factors are scalable, and a dose-response clinical interpretation should be considered. Secondly, the behavioural risk factors were assessed based on self-report, which may result in under- or over-estimation of certain behaviours. A known limitation of FFQs used in nutritional epidemiology is the imperfect measure of diet.^(44,45)^ Using the Fung *et al.* scoring method to calculate the DASH diet score using quintiles of food group intake, we have minimised misclassification that may be present due to measurement error associated with a FFQ.^(21)^ For this population-based scoring method that is characterised by overall dietary pattern, an FFQ is the best available tool, particularly when compared to seven-day food records or 24-hour dietary recalls that may not capture the variety of food intake that characterises the DASH dietary pattern. Finally, the MASALA cohort is comprised of participants with high educational attainment and income levels, consistent with early U.S. immigration patterns for South Asians, limiting the generalizability to South Asians with lower educational attainment or income. Recruitment of more diverse South Asian populations for follow-up examinations is currently underway.

Our study has several strengths. The MASALA study is the first multi-centre community-based cohort of South Asian adults living in the U.S., with data collection performed incorporating culturally competent practices.^(13,46)^ The Typical Week’s Physical Activity Questionnaire^(15)^ was able to provide information on both physical activity and sedentary behaviour, which were used in the derived behavioural risk factors score. Dietary data was collected using an interviewer-administered FFQ validated for South Asians living in North America, which included several culturally appropriate South Asian foods and beverages.^(18)^ This allowed us to compute a DASH diet score using the raw data from the FFQ.

## Conclusion

Our findings emphasise the relevance of focusing on several health behaviours in combination with one another to holistically address their impact on hypertension and ASCVD risk, particularly among South Asian immigrants. We conclude from our study that South Asians with more behavioural risk factors for hypertension followed a diet that was less consistent with the recommended DASH dietary pattern. Clinicians should consider the impact of several lifestyle behaviours on one another and counsel patients accordingly to help mitigate overall risk for hypertension and ASCVD. Policies and targeted interventions should incorporate several behavioural factors to increase the potential impact on overall health.

## Supporting information

Hussain et al. supplementary material 1 Hussain et al. supplementary material

Hussain et al. supplementary material 2 Hussain et al. supplementary material

Hussain et al. supplementary material 3 Hussain et al. supplementary material

Hussain et al. supplementary material 4 Hussain et al. supplementary material

Hussain et al. supplementary material 5 Hussain et al. supplementary material

## Supplementary material

The supplementary material for this article can be found at https://doi.org/10.1017/jns.2025.8
