# Supplementary material for: Association between behavioural risk factors for hypertension and concordance with the Dietary Approaches to Stop Hypertension dietary pattern among South Asians in the Mediators of Atherosclerosis in South Asians Living in America (MASALA) study
Source: J Nutr Sci. 2025 Mar 5;14:e22. doi: 10.1017/jns.2025.8 (PMC11894414; doi:10.1017/jns.2025.8)
Supplement: Hussain et al. supplementary material 1 — Hussain et al. supplementary material [file S2048679025000084sup001.docx]

| **Supplementary Table 1. Scoring criteria for the DASH-Style diet and mean intake for Q1 (low consumption) and Q5 (high consumption) among South Asians in the MASALA study (n=871).** | | | | |
| --- | --- | --- | --- | --- |
| **Component** | **Foods** | **Scoring Criteria** | **Q1, Servings/d** | **Q5, Servings/d** |
| Fruit | Apple/Pear; Citrus Fruit; Banana; Grapes; Peach/Nectarine/Plum; Melon; Tropical Fruit | Q1 = 1 point  Q2 = 2 points  Q3 = 3 points  Q4 = 4 points  Q5 = 5 points | 0.6 | 4.1 |
| Vegetables | Bell Peppers; French/String Benas; Broccoli; Cauliflower; Cabbage; Carrots; Celery; Dark Leafy Green Vegetables; Mushrooms; Okra/Bhindi; Onion; Summer Squash/Zucchini; Tomato; Vegetable Kofta; Mixed Vegetable Salad/Stir Fry; Root Vegetables |  | 1.6 | 7.7 |
| Whole Grains | Whole Wheat Bread/Roll (100%, incl. dark rye; 60% incl light rye); Roti/Chapati; Bran Granola Cereals; Whole Wheat Cereals; Cooked Cereal (i.e. porridge, oatmeal, dalia, bulgar); Brown Rice; Quinoa |  | 0.6 | 3.5 |
| Low-fat Dairy | Milk (including Skim, 1%, 2%, and lactaid); Part-Skim cheese; Low-Fat Plain Yogurt |  | 0.1 | 2.9 |
| Nuts and Legumes | Peas/Matar; Lentils; Dal; Sambhar/Rasam; Chickpeas; Other Dried Beans; Nuts; Tofu; Peanut Butter |  | 0.7 | 3.6 |
| Sodium^a^ | Sum of sodium content of all foods in FFQ | Q1 = 5 points  Q2 = 4 points  Q3 = 3 points  Q4 = 2 points  Q5 = 1 point | 1,573 mg | 4,285 mg |
| Sugar-Sweetened Beverages^a^ | Non-Diet Cola; Clear Soft Drinks (i.e. 7-up/Sprite); Orange/Grapefruit Juice; Apple/Pineapple Juice; Fruit Drink; Iced Tea; Lemonade; Yogurt Drink (i.e. Lassi with sugar) |  | 0.003 | 1.4 |
| Red and Processed Meats^a^ | Beef; Pork; Goat/Lamb; Pork Sausage; Ham; Salami; Bologna; Bacon; Liver; Fried Chicken Nuggets |  | 0 | 0.7 |
| ^a^ Higher quintiles represent higher intake. In constructing the DASH score, higher intake (higher quintiles) of these components receive lower scores | | | | |
