# Supplementary material for: Association between behavioural risk factors for hypertension and concordance with the Dietary Approaches to Stop Hypertension dietary pattern among South Asians in the Mediators of Atherosclerosis in South Asians Living in America (MASALA) study
Source: J Nutr Sci. 2025 Mar 5;14:e22. doi: 10.1017/jns.2025.8 (PMC11894414; doi:10.1017/jns.2025.8)
Supplement: Hussain et al. supplementary material 2 — Hussain et al. supplementary material [file S2048679025000084sup002.docx]

| **Supplementary Table 2. Age-adjusted and multivariable-adjusted DASH diet score by smoking status (never versus current/former), among South Asian adults in the MASALA study (n=871).** | | | | |
| --- | --- | --- | --- | --- |
|  | Never Smoker (n=725) | Current/Former Smoker  (n=146) | | *P_trend_** |
|  | *Reference* | β/OR (SE) | 95% CI |  |
| DASH Diet Score (continuous) |  |  |  |  |
| Age Adjusted | 0.00 | -2.39 (0.39) | -3.15, -1.64 | <0.0001 |
| Model 1^+^ | 0.00 | -1.28 (0.40) | -2.07, -0.49 | 0.001 |
| Model 2^++^ | 0.00 | -1.25 (0.40) | -2.03, -0.48 | 0.002 |
| DASH Diet Score (Low (13-20) vs. Medium (21-28)) | |  |  |  |
| Age Adjusted | 1.00 | 0.48 (0.10) | 0.32, 0.74 | 0.001 |
| Model 1^+^ | 1.00 | 0.76 (0.19) | 0.47, 1.23 | 0.27 |
| Model 2 ^++^ | 1.00 | 0.77 (0.19) | 0.47, 1.24 | 0.28 |
| DASH Diet Score (Low (13-20) vs. High (29-35)) | |  |  |  |
| Age Adjusted | 1.00 | 0.25 (0.08) | 0.13, 0.47 | <0.0001 |
| Model 1^+^ | 1.00 | 0.53 (0.19) | 0.25, 1.05 | 0.07 |
| Model 2 ^++^ | 1.00 | 0.51 (0.19) | 0.25, 1.05 | 0.07 |
| SE: Standard Error; CI: Confidence Interval  *p-trend calculated using smoke category (current, former, never) as a continuous covariate.  ^+^Model 1: Adjusted for age, gender (men/women), percent life lived in the U.S., education (≥Bachelors/<Bachelors), physical activity (ideal, intermediate, poor), TV watching (≥1 hour/week vs. <1 hour/week), alcohol intake (<1 drink/week vs. ≥1 drink/week), acculturation (assimilation, separation, integration)  ^++^Model 2: Model 1 + energy (kcal/d) | | | | |
