# Supplementary material for: Association between behavioural risk factors for hypertension and concordance with the Dietary Approaches to Stop Hypertension dietary pattern among South Asians in the Mediators of Atherosclerosis in South Asians Living in America (MASALA) study
Source: J Nutr Sci. 2025 Mar 5;14:e22. doi: 10.1017/jns.2025.8 (PMC11894414; doi:10.1017/jns.2025.8)
Supplement: Hussain et al. supplementary material 4 — Hussain et al. supplementary material [file S2048679025000084sup004.docx]

| **Supplementary Table 4. Age-adjusted and multivariable-adjusted DASH diet score by physical activity level (poor, intermediate, ideal), among South Asian adults in the MASALA study (n=871).** | | | | | | |
| --- | --- | --- | --- | --- | --- | --- |
|  | Poor  (n=134) | Intermediate  (n=171) | | Ideal  (n=566) | | *P_trend_** |
|  | *Reference* | β/OR (SE) | 95% CI | β/OR (SE) | 95% CI |  |
| DASH Diet Score (continuous) |  |  |  |  |  |  |
| Age Adjusted | 0.00 | 0.71 (0.49) | -0.26, 1.67 | 2.13 (0.41) | 1.32, 2.93 | <0.0001 |
| Model 1^+^ | 0.00 | 0.67 (0.46) | -0.24, 1.59 | 2.21 (0.40) | 1.44, 2.99 | <0.0001 |
| Model 2^++^ | 0.00 | 0.76 (0.46) | -0.14, 1.66 | 2.23 (0.39) | 1.46, 2.99 | <0.0001 |
| DASH Diet Score (Low (13-20) vs. Medium (21-28)) | |  |  |  |  |  |
| Age Adjusted | 1.00 | 1.09 (0.29) | 0.64, 1.84 | 2.00 (0.47) | 1.27, 3.16 | 0.001 |
| Model 1^+^ | 1.00 | 1.14 (0.33) | 0.65, 2.01 | 2.60 (0.68) | 1.56, 4.33 | <0.0001 |
| Model 2 ^++^ | 1.00 | 1.16 (0.68) | 0.66, 2.04 | 2.61 (0.68) | 1.57, 4.33 | <0.0001 |
| DASH Diet Score (Low (13-20) vs. High (29-35)) | |  |  |  |  |  |
| Age Adjusted | 1.00 | 1.62 (0.65) | 0.74, 3.56 | 4.18 (1.44) | 2.13, 8.20 | <0.0001 |
| Model 1^+^ | 1.00 | 1.79 (0.76) | 0.78, 4.09 | 6.02 (2.26) | 2.88, 12.55 | <0.0001 |
| Model 2 ^++^ | 1.00 | 1.85 (0.79) | 0.80, 4.28 | 6.15 (2.32) | 2.94, 12.88 | <0.0001 |
| SE: Standard Error; CI: Confidence Interval  *p-trend calculated by unadjusted linear regression, using total physical activity score (1-3) as a continuous covariate.  ^+^Model 1: Adjusted for age, gender (men/women), percent life lived in the U.S., education (≥Bachelors/<Bachelors), smoking status (former/current vs. never), TV watching (≥1 hour/week vs. <1 hour/week), smoking status (current/former vs. never), acculturation (assimilation, separation, integration)  ^++^Model 2: Model 1 + energy (kcal/d) | | | | | | |
