# Supplementary material for: Association between behavioural risk factors for hypertension and concordance with the Dietary Approaches to Stop Hypertension dietary pattern among South Asians in the Mediators of Atherosclerosis in South Asians Living in America (MASALA) study
Source: J Nutr Sci. 2025 Mar 5;14:e22. doi: 10.1017/jns.2025.8 (PMC11894414; doi:10.1017/jns.2025.8)
Supplement: Hussain et al. supplementary material 5 — Hussain et al. supplementary material [file S2048679025000084sup005.docx]

| **Supplementary Table 5. Age-adjusted and multivariable-adjusted DASH diet score by TV watching (<1 hour/week versus ≥1 hour/week), among South Asian adults in the MASALA study (n=871).** | | | | |
| --- | --- | --- | --- | --- |
|  | ≤1 hour/week (n=525) | >1 hour/week  (n=346) | | *P_trend_** |
|  | *Reference* | β/OR (SE) | 95% CI |  |
| DASH Diet Score (continuous) |  |  |  |  |
| Age Adjusted | 0.00 | -0.27 (0.31) | -0.88, 0.33 | 0.06 |
| Model 1^+^ | 0.00 | -0.22 (0.29) | -0.78, 0.34 | 0.44 |
| Model 2^++^ | 0.00 | -0.30 (0.28) | -0.85, 0.26 | 0.29 |
| DASH Diet Score (Low (13-20) vs. Medium (21-28)) | |  |  |  |
| Age Adjusted | 1.00 | 0.82 (0.16) | 0.56, 1.19 | 0.42 |
| Model 1^+^ | 1.00 | 0.84 (0.17) | 0.57, 1.25 | 0.40 |
| Model 2 ^++^ | 1.00 | 0.84 (0.17) | 0.57 1.25 | 0.40 |
| DASH Diet Score (Low (13-20) vs. High (29-35)) | |  |  |  |
| Age Adjusted | 1.00 | 0.88 (0.21) | 0.56, 1.39 | 0.19 |
| Model 1^+^ | 1.00 | 0.91 (0.23) | 0.56, 1.49 | 0.70 |
| Model 2 ^++^ | 1.00 | 0.88 (0.22) | 0.53, 1.44 | 0.60 |
| SE: Standard Error; CI: Confidence Interval  *p-trend calculated using continuous minutes of TV watching per week covariate  ^+^Model 1: Adjusted for age, gender (men/women), percent life lived in the U.S., education (≥Bachelors/<Bachelors), smoking status (former/current vs. never), physical activity (poor, intermediate, ideal), smoking status (current/former vs. never), acculturation (assimilation, separation, integration)  ^++^Model 2: Model 1 + energy (kcal/d) | | | | |
